# Polymorphisms of the *MxA* and *MxB* genes are associated with biochemical indices and viral subtypes in Yunnan HCV patients

**DOI:** 10.3389/fcimb.2023.1119805

**Published:** 2023-01-19

**Authors:** Mengzhu He, Min Liu, Jiawei Geng, Li Liu, Peng Huang, Ming Yue, Xueshan Xia, A-Mei Zhang

**Affiliations:** ^1^ Faculty of Life Science and Technology, Kunming University of Science and Technology, Kunming, Yunnan, China; ^2^ Department of Infectious Diseases, The First People's Hospital of Yunnan Province, Yunnan, China; ^3^ School of Public Health, Nanjing Medical University, Nanjing, China; ^4^ Kunming Medical University, Yunnan, China

**Keywords:** HCV infection, biochemical indices, subtype, *MxA*, *MxB*

## Abstract

**Introduction:**

Hepatitis C virus (HCV) infection was the primary reason causing critical hepatic Q7 diseases. Although direct-acting antiviral agents (DAA) were widely used in clinics, anti-drug mutation, the outcome of patients with different viral subtypes, and recurrence suggested that HCV pathogenic mechanism should be studied further. HCV infection, replication, and outcome were influenced by the IFNL4 and itsdownstream genes (MxA and MxB). However, whether genetic polymorphisms of these genes played necessary roles required verification in the Yunnan population.

**Methods and Results:**

After analyzing the genotypes and allele frequencies of seven single nucleotide polymorphisms (SNP), we found the association between the genotype and allele frequencies of rs11322783 in the IFNL4 gene and HCV infection in Yunnan population. Furthermore, the genetic polymorphisms of the MxA and MxB genescould influence liver function of HCV patients. The indirect bilirubin (IBIL) and albumin (ALB) levels showed significant differences among HCV patients, who carried various genotypes. The IBIL levels were associated with genotypes of rs17000900 (P= 0.025) and rs2071430 (P= 0.037) in the MxA gene, and ALB levels were associated with genotypes of rs2838029 (P= 0.010) in the MxB gene. Similarly, the genotypes of SNPs also showed significant difference in patients infected with subtype 3a (P=0.035) and 2a (P=0.034). However, no association was identified between expression level and SNPs of the MxA and MxB genes. Furthermore, HCV subtype 3b was found to be the predominantly epidemic strain in Yunnan Province.

**Conclusion:**

In conclusion, the association between biochemical indices/HCV subtypes and SNPs in the MxA and MxB genes was identified in Yunnan HCV population.

## Introduction

Hepatitis C virus (HCV) infection could lead to critical hepatitis diseases, such as cirrhosis and hepatocellular carcinoma (HCC). Although direct-acting antivirals (DAAs) drugs are used worldwide, approximately 1.75 million new HCV infections occurred in 2015 (W.H.O, 2017). The mortality trends of infectious hepatitis disease were increasing from 2000 to 2015, differing from other popular infectious diseases, such as human immunodeficiency virus (HIV), tuberculosis (TB), and malaria. Thus, the pathogenic mechanisms of HCV infection should be studied further.

Interferons (IFNs) are the common used drugs for HCV infection therapy, and they could active Jak/STAT signaling pathway and further induce some interferon-stimulated genes (ISGs) (Heim and Thimme, 2014). The expression and function of Interferon lambda 4 (*IFNL4*) gene is controlled by its genetically transcriptional regulation (Zhou et al., 2020). The *MyXovirus resistance* (*Mx*) genes belongs to the ISGs family. Firstly, the *MyXovirus resistance 1* (*MxA* or *Mx1*) gene was identified as one of the main protected factors for mice against influenza viruses (Lindenmann, 1964). Then, it is investigated that the *MxA* gene belonged to natural immune system and had wide-spectra anti-virus activity, which is induced by interferon (Haller et al., 2018). Although the antiviral activity of the *MxB* gene is limited compared to the *MxA* gene, it could interfere with HCV RNA replication by interacting with the NS5A protein (Yi et al., 2019). These reports suggested the *Mxs* genes were necessary natural factors for inhibiting HCV in patients.

To date, the genetic polymorphisms of the *MxA* gene have been reported to be associated with HCV infection or clearance of those with HCV infection (Garcia-Alvarez et al., 2017), but whether single nucleotide polymorphisms (SNPs) of the *MxB* gene could influence HCV infection, biochemical characteristics, or outcome of HCV patients are not clear. In this study, the SNPs of the *MxA* and *MxB* genes in HCV patients and controls from Yunnan Province were analyzed.

## Materials and methods

### Individuals and clinical data

We recruited 347 patients with chronic HCV infection and 448 normal controls from the First People’s Hospital of Yunnan Province from 2019 to 2022. 3 mL of whole blood was collected from each individual. The patients were diagnosed as chronic HCV infected persons by the symptoms and liver function test. All HCV-infected patients were identified to be anti-HCV positive by HCV ELISA Kit (ORTHO, USA), and all patients were without any medical treatment when we collected the samples. All patients were identified without serious liver disease, such as fibrosis, cirrhosis and hepatocellular carcinoma). The basic information and liver function data of all samples [including alanine transaminase (ALT), aspartate transaminase (AST), total bilirubin (TBIL), direct bilirubin (DBIL), indirect bilirubin (IBIL), total protein (TP), albumin (ALB), globin (GLOB)] of HCV patients were collected for further analysis. Based on the clinical phenotype and biochemical indexes, all chronic HCV infected patients were identified without Hepatitis B virus (HBV) or HIV infection by using Quantitative CLIA Kit (Autobio, China) and Anti-HIV ELISA Kit (WANTAI, China). Written informed consents of the Declaration of Helsinki were obtained from all participants prior to the study. The institutional review board of Kunming University of Science and Technology approved this study.

### HCV RNA loading quantification and genotyping

HCV RNA was abstracted from 100 µL serum of patients by using TIANamp Virus RNA Kit (TIANGEN, China), and viral RNA load was quantified by using the primers and probe in our previous study (Song et al., 2019). The detection limit was set at 1,000 copies/mL. HCV RNA was further transformed as log10‐transformation. The NS5B gene of HCV was genotyped in each patient by using the method described in our previous study (Zhang et al., 2014).

### SNP selection and genotyping

Genomic DNA was extracted from whole blood by using the Blood Genomic DNA Miniprep Kit (Axygen, USA). Seven tagSNPs were genotyped by using SnapShot method according to previous study (Zheng et al., 2022), including two SNPs (rs11322783 and rs117648444) in the *IFNL4* gene, two SNPs (rs2071430 and rs17000900) in the *MxA* gene and three SNPs (rs9982944, rs408825, and rs2838029) in the *MxB* gene,. Then, 10% of the genotyping results were further identified by using Sanger sequencing method.

### RNA expression level detection

Total RNA was extracted from 200 µL whole blood of HCV patients using the RNAsimple Total RNA Kit (TIANGEN, China). Then, the level of RNA expression of the *MxA* and *MxB* genes was detected using fluorescent quantitative real-time PCR. The primers for quantifying target genes were GTGCATTGCAGAAGGTCAGA/CTGGTGATAGGCCATCAGGT (for the *MxA* gene) and CAGAGGCAGCGGAATCGTAA/TGAAGCTCTAGCTCGGTGTTC (for the *MxB* gene). The *GAPDH* gene was used as the reference gene, and the primers for quantification were GGCATCCTGGGCTACACTGAG/CATACCAGGAAATGAGCTTGAC. The ChamQ SYBR^®^ qPCR Master Mix (Vazyme, China) and Quant Gene 9600 were used to determine the RNA expression levels of the *MxA* and *MxB* genes of HCV patients.

### Data analysis

The genotype and allele frequencies between HCV patients and controls are analyzed by using Chi-square text. A student t test (two tailed) was used to compare viral load between two HCV subtypes, biochemical indices among patients with different genotypes, and RNA level expression of *MxA* and *MxB* genes. The correlation analysis was used to determine the relationship between viral load of HCV and RNA level of the *MxA* or *MxB* gene (two-sided). A *P*-value less than 0.05 is considered statistically significant.

## Results

The gender ratio (male: female) was similar between HCV patient group (1.4:1) and controls (1.6:1). There were 201 males and 146 females in the HCV patients, and 275 males and 173 females in the control group. The mean age was 44.70 ± 0.89 and 40.58 ± 0.53 years in the HCV patients and controls, respectively. The genotype and allele frequencies, excluding SNP rs11322783, showed no significant difference between HCV patients and controls. Genotype ΔG/T of rs11322783 showed significantly lower frequency in HCV patients (0%) than that in controls (5.58%); however, the frequency of genotype TT was statistically higher in HCV patients (99.42%) than that in controls (94.20%) (**Table 1**). Similarly, allele ΔG of rs11322783 was a protective factor for HCV infection in Yunnan, with a frequency of 0.58% (4/694) and 3.01% (27/896) in patients and controls, respectively. This result suggested genotype and allele of rs11322783 could influence HCV infection in the Yunnan population.

**Table 1:** Frequencies of SNPs in the *IFNL4*, *MxA*, and *MxB* gene in Yunnan HCV patients and controls.

SNP genotype/allele	HCV patients (N=347)	Controls (N=448)	*P*-value	OR (95% CI)
rs11322783 (*IFNL4*)				
GenotypeΔG/ΔG	2	1	0.584	2.591 (0.300-37.63)
ΔG/T	0	25	<0.0001	0.0001 (0-0.165)
TT	345	422	<0.0001	10.63 (2.695-45.68)
Allele ΔG	4	27	0.0004	0.187 (0.070-0.517)
T	690	869		5.360 (1.936-14.32)
rs117648444 (*IFNL4*)				
Genotype AA	0	0	–	–
AG	6	7	0.922	1.109 (0.368-3.025)
GG	341	441	0.922	0.902 (0.331-2.718)
Allele A	6	7	0.922	1.108 (0.371-3.018)
G	688	889		0.903 (0.331-2.699)
rs2071430 (*MxA*)				
Genotype GG	162	217	0.675	0.932 (0.706-1.229)
GT	150	188	0.776	1.053 (0.793-1.396)
TT	35	43	0.913	1.057 (0.656-1.669)
Allele G	474	622	0.672	0.949 (0.757-1.178)
T	220	274		1.054 (0.849-1.305)
rs17000900 (*MxA*)				
Genotype AA	6	13	0.401	0.589 (0.229-1.457)
AC	91	114	0.867	1.041 (0.753-1.431)
CC	250	321	0.966	1.020 (0.745-1.387)
Allele A	103	140	0.719	0.941 (0.716-1.239)
C	591	756		1.063 (0.807-1.397)
rs9982944 (*MxB*)				
Genotype AA	29	40	0.876	0.930 (0.555-1.552)
AG	159	222	0.331	0.861 (0.652-1.135)
GG	159	186	0.253	1.191 (0.900-1.578)
Allele A	217	302	0.330	0.895 (0.724-1.103)
G	477	594		1.118 (0.907-1.382)
rs408825 (*MxB*)				
Genotype CC	11	18	0.659	0.782 (0.354-1.619)
CT	119	131	0.149	1.263 (0.940-1.696)
TT	217	299	0.247	0.832 (0.622-1.114)
Allele C	141	167	0.438	1.113 (0.870-1.431)
T	553	729		0.899 (0.699-1.149)
rs2838029 (*MxB*)				
Genotype AA	2	5	0.477	0.514 (0.102-2.390)
AG	51	58	0.543	1.159 (0.765-1.739)
GG	294	385	0.705	0.908 (0.618-1.348)
Allele A	55	68	0.878	1.048 (0.727-1.520)
G	639	828		0.954 (0.658-1.376)

347 chronic HCV infected persons and 448 controls were used to analyze genotypes and allele frequencies of seven SNPs.

The biochemical data was analyzed among 270 HCV patients with different genotypes of each SNP (**Table 2**). The IBIL levels showed a significant difference among various genotypes of two SNPs in the *MxA* gene of HCV patients. In HCV patients with genotype AC of rs17000900 or genotype GT of rs2071430, the IBIL level were significantly higher than that in the other patients. The ALB levels were much lower in HCV patients carried genotype AA and AG of rs2838029 than other patients. These results suggested genetic polymorphisms of the *MxA* and *MxB* genes could influence the liver function of HCV patients.

**Table 2:** Biochemical indexes analysis among HCV patients with various SNP genotypes.

Biochemical features (unit)	rs17000900				rs2071430			
	AA	AC	CC	*P*- value	GG	GT	TT	*P*- value
AST (U/L)	88.67 ± 21.70	10.7.4 ± 30.30	72.11 ± 7.05	0.271	76.11 ± 10.48	90.14 ± 19.53	76.13 ± 8.80	0.775
ALT (U/L)	115.2 ± 23.16	124.0 ± 28.82	91.13 ± 9.71	0.371	92.42 ± 14.29	105.5 ± 18.69	115.4 ± 14.95	0.742
TBIL (µmol/L)	15.60 ± 4.03	28.06 ± 5.61	19.97 ± 2.37	0.262	19.24 ± 1.99	27.11 ± 4.74	13.87 ± 1.43	0.120
DBIL (µmol/L)	7.27 ± 2.22	14.71 ± 4.18	10.57 ± 1.86	0.533	9.86 ± 1.57	14.82 ± 3.62	6.23 ± 0.69	0.228
IBIL (µmol/L)	8.33 ± 1.83	13.35 ± 1.81	9.47 ± 0.57	0.025	9.49 ± 0.56	12.29 ± 1.34	7.64 ± 0.78	0.037
TP (g/L)	74.33 ± 3.05	74.29 ± 0.91	73.98 ± 0.46	0.940	74.14 ± 0.55	73.93 ± 0.69	74.36 ± 1.23	0.944
ALB (g/L)	39.17 ± 2.44	40.85 ± 0.71	40.72 ± 0.40	0.781	40.63 ± 0.52	40.78 ± 0.52	40.86 ± 1.04	0.971
GOLB (g/L)	35.17 ± 2.60	33.47 ± 0.68	33.34 ± 0.41	0.737	33.63 ± 0.55	33.20 ± 0.50	33.43 ± 1.03	0.845
Biochemical features (unit)	rs9982944				rs408825			
	AA	AG	GG	*P*- value	CC	CT	TT	*P*- value
AST (U/L)	74.65 ± 10.88	85.78 ± 17.83	79.85 ± 11.04	0.929	77.33 ± 17.05	100.6 ± 23.89	72.00 ± 7.98	0.379
ALT (U/L)	97.77 ± 17.90	102.5 ± 17.06	99.30 ± 15.07	0.986	83.67 ± 17.47	115.1 ± 22.52	93.46 ± 11.17	0.595
TBIL (µmol/L)	15.65 ± 2.19	23.52 ± 4.21	21.83 ± 2.41	0.640	20.09 ± 3.07	27.72 ± 5.77	19.10 ± 1.76	0.202
DBIL (µmol/L)	7.11 ± 1.11	12.84 ± 3.26	11.21 ± 1.78	0.655	9.88 ± 2.39	16.50 ± 4.62	9.04 ± 1.11	0.129
IBIL (µmol/L)	8.54 ± 1.18	10.74 ± 1.09	10.66 ± 0.85	0.639	10.21 ± 1.03	11.22 ± 1.25	10.14 ± 0.78	0.731
TP (g/L)	74.13 ± 1.30	74.23 ± 0.60	73.89 ± 0.63	0.922	75.11 ± 2.48	73.56 ± 0.76	74.30 ± 0.49	0.621
ALB (g/L)	39.48 ± 1.23	40.96 ± 0.46	40.71 ± 0.56	0.509	40.22 ± 2.07	40.11 ± 0.60	41.09 ± 0.43	0.403
GOLB (g/L)	34.61 ± 1.16	33.24 ± 0.49	33.38 ± 0.55	0.562	34.78 ± 2.74	33.51 ± 0.658	33.30 ± 0.40	0.733
Biochemical features (unit)	rs2838029							
	AA & AG	GG		*P*- value				
AST (U/L)	106.6 ± 42.92	77.25 ± 7.90		0.961				
ALT (U/L)	112.5 ± 39.99	98.30 ± 9.73		0.794				
TBIL (µmol/L)	25.73 ± 5.53	21.40 ± 2.50		0.517				
DBIL (µmol/L)	14.90 ± 4.57	11.05 ± 1.89		0.581				
IBIL (µmol/L)	10.83 ± 1.26	10.45 ± 0.732		0.599				
TP (g/L)	73.31 ± 1.33	74.22 ± 0.42		0.411				
ALB (g/L)	38.88 ± 0.90	41.07 ± 0.37		0.010				
GOLB (g/L)	34.40 ± 1.11	33.23 ± 0.36		0.498				

Biochemical indices were analyzed in 270 HCV patients with different genotypes of each SNP. The indices were expressed by mean ± SE.

**Figure 1:**
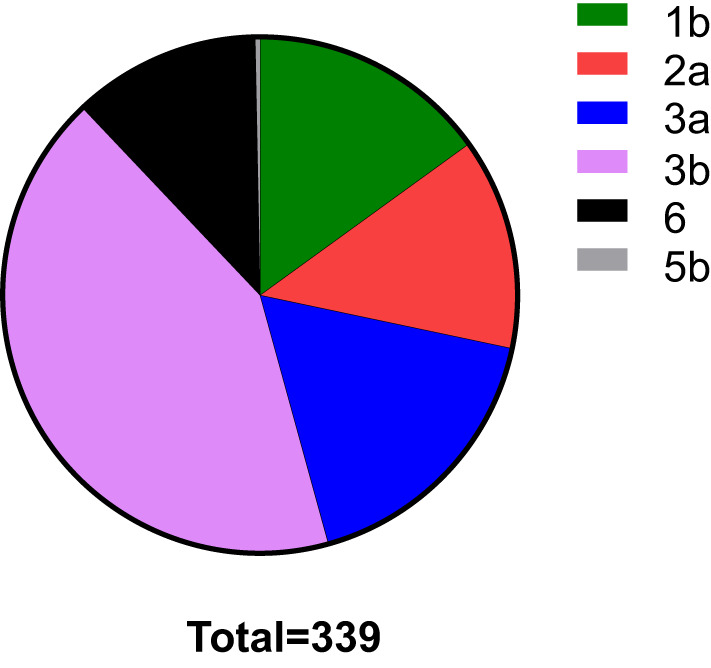
The distribution of HCV subtypes in this study. Totally 339 HCV patients were used to detect HCV subtypes. The subtype 3b was dominant in Yunnan Province.

**Figure 2:**
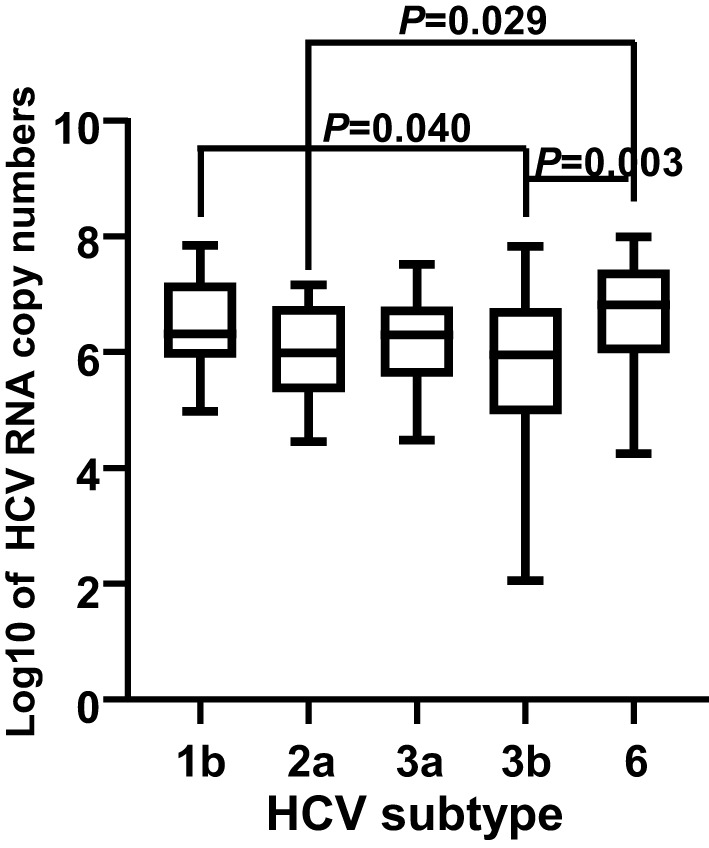
Comparison of viral load among 137 HCV patients with different subtypes. The viral loads were transformed into log10 of the copy numbers. A *P* value less than 0.05 was considered as significant difference.

In total, HCV subtypes were determined in 339 HCV patients (**Figure 1**). Subtype 3b was the main subtype in Yunnan HCV patients occurring in 42.18% of individuals (n= 143). The frequencies of HCV subtype 3a (n= 59, 17.40%), 1b (n= 51, 15.04%), 2a (n= 45, 13.27%), and 6 (n= 40, 11.80%) were similar in Yunnan population. Only one sample belonged to subtype 5b. Although HCV viral load was detected in only 137 patients, the viral load was compared among HCV patients with different subtypes (**Figure 2**). The results showed significantly higher viral load existed in the patients with subtype 6 than in patients with subtype 2a (*P*= 0.029) or 3b (*P*= 0.003). The viral load was statistically higher in the patients with subtype 1b than those with subtype 3b. These results suggested that the viral load of HCV patients might be affected by HCV subtypes.

The frequencies distribution of subtypes in HCV patients with different genotypes of each SNP in the *MxA* and *MxB* genes were analyzed (**Table 3**). The frequency of subtype 3a was lower in patients carried genotype GG of rs9982944 (11.69%, 18/154) than in those with genotype AA (25%, 7/28) or AG (22.08%, 34/154). The frequency of subtype 2a was significantly lower in HCV patients with genotype AG of rs408825 (6.84%, 8/117) than in those with genotype AA (16.98%, 36/212) or GG (11.11%, 1/9). These results suggested the possible protective role of genotype GG of rs9982944 and genotype AG of rs408825 against HCV subtype 3b and 2a infection, respectively.

**Table 3:** HCV subtypes frequencies in patients carried with different SNP genotypes.

HCV subtypes	SNP Genotypes			*P*-value
	rs2071430			
	GG	GT	TT	
1b	29	20	2	0.150
2a	24	19	2	0.335
3a	25	28	6	0.695
3b	66	58	19	0.310
6	15	19	6	0.354
	rs17000900			
	AA	AC	CC	
1b	0	11	40	0.362
2a	0	9	36	0.318
3a	0	19	40	0.325
3b	5	37	101	0.121
6	1	14	25	0.396
	rs9982944			
	AA	AG	GG	
1b	3	20	28	0.334
2a	2	18	25	0.288
3a	7	34	18	0.035
3b	11	61	71	0.434
6	5	23	12	0.098
	rs408825			
	GG	AG	AA	
1b	2	19	30	0.732
2a	1	8	36	0.034
3a	1	21	37	0.873
3b	5	56	82	<0.0001
6	0	13	27	0.489
	rs2838029			
	AA	AG	GG	
1b	0	11	40	0.256
2a	0	4	41	0.434
3a	0	10	49	0.688
3b	2	21	120	0.251
6	0	3	37	0.349

HCV subtypes were studied in 339 HCV patients with different genotypes of each SNP.

When comparing the RNA expression level of the *MxA* gene and *MxB* gene in the patients with various genotypes of each SNP of the corresponding gene (**Table 4**), no significant difference was identified. Similarly, no correlation was identified between the RNA expression level of the *MxA* gene (r= 0.083, *P*= 0.343) or the *MxB* gene (r= 0.036, *P*= 0.691) and the viral load of HCV patients.

**Table 4:** RNA expressing level of the *MxA* and *MxB* gene in HCV patients with various genotypes of these two genes.

Gene	RNA expressing level in various genotypes			*P*-value
rs17000900	AA	AC	CC	
*MxA*	3.461 ± 0.696	3.076 ± 0.321	2.573 ± 0.225	0.386
rs2071430	GG	GT	TT	
*MxA*	2.541 ± 0.296	2.930 ± 0.210	3.244 ± 0.455	0.332
rs9982944	AA	AG	GG	
*MxB*	7.401 ± 0.910	6.511 ± 0.374	6.209 ± 0.442	0.495
rs408825	CC	CT	TT	
*MxB*	7.022 ± 2.344	6.463 ± 0.387	6.460 ± 0.375	0.955
rs2838029	AA	AG	GG	
*MxB*	–	6.372 ± 0.508	6.495 ± 0.312	0.874

## Discussion

Although over 90% of HCV patients could be cured by using DAA, some were still treated by using IFN-α combined Ribavirin owing to the high price of DAA and anti-drug mutation of DAA. Thus, the mechanism of IFN against HCV was necessary to further study. IFN-α could activate the Jak-STAT signaling pathway and further induce MxA production, which could inhibit HCV replication (Stevenson et al., 2011). The MxB belongs to the same family as MxA and plays wide anti-virus roles. However, reports regarding how MxB inhibited HCV replication were few. Li et al. found that MxB competitively interacted with NS5A of HCV and reduced the combination between NS5A and CypA, which provided formation of HCV infection (Li et al., 2022). These results suggested the importance of MxA and MxB in inhibiting HCV infection and replication.

The genetic polymorphisms of the *MxA* gene were considered to influence HCV infection, viral clearance, and outcome in many studies (Hassany et al., 2017; Zang et al., 2018), but no association study was performed between SNPs of the *MxB* gene and Chinese HCV patients. In this study, although no association was identified between SNPs of the *MxA* and *MxB* genes with HCV infection in the Yunnan population, HCV subtypes and biochemical indices demonstrated significant differences among patients with various genotypes. Zang et al. reported that genotype TT of rs2071430 in the *MxA* gene preferred to induce patients into chronic HCV infection (Zang et al., 2018). In Egyptian and Japanese HCV patients, the promoter SNPs of the *MxA* gene were considered independently influenced factors in response to IFN therapy (Suzuki et al., 2004; Hassany et al., 2017).

In liver allograft patients with or without HCV infection, the expression level of the *MxA* gene was positively associated with AST levels (>40 U/L) in patients’ liver biopsies (Borgogna et al., 2009). SNP at nt-88 of the *MxA* gene was identified to be an independent determining factor for outcome of IFN therapy (Suzuki et al., 2004). Similarly, genotype TT of rs2071430 in the *MxA* gene influenced HCV infection chronicity of patients (Zang et al., 2018). Similarly, we found that genotype GT of rs2071430 was associated with high IBIL levels of patients. These suggested that genetic polymorphisms of the *MxA* gene might influence the pathogenic progress and outcome of patients. To the best of our knowledge, this was the first study to identify the association between genetic polymorphisms of the *MxB* gene and biochemical indices and subtypes of HCV patients.

The genotypes of rs11322783 in the *IFNL4* gene were widely reported as an important host genetic factor influencing viral clearance and outcome of HCV patients (Aka et al., 2014; O'Brien et al., 2015). The allele ΔG and T of rs11322783 were found to play protective and risk roles in HCV infection. However, we could not analyze the association between genotypes of rs11322783 and biochemical indices of patients owing to the few numbers of patients with genotype ΔG/ΔG. These studies suggested that SNPs of the *IFNL4* gene could influence HCV infection, disease progress, and treatment effect.

In summary, we firstly identified that genetic polymorphisms of the *MxA* and *MxB* genes were associated with biochemical indices and HCV subtypes of patients in Yunnan.

## Data availability statement

The datasets presented in this study can be found in online repositories. The names of the repository/repositories and accession number(s) can be found in the article/**Supplementary Material**.

## Ethics statement

The studies involving human participants were reviewed and approved by The institutional review board of Kunming University of Science and Technology approved this study. The patients/participants provided their written informed consent to participate in this study.

## Author contributions

A-MZ and XX designed this study. MH and ML performed the experiments. JG, PH and MY collected the samples. A-MZ and LL analyzed the data. A-MZ and MH prepared the manuscript. All authors contributed to the article and approved the submitted version.
